# Bias of Odds Ratio Estimate in Fisher's Exact Test

**DOI:** 10.1002/mpr.70076

**Published:** 2026-06-27

**Authors:** Xiaofeng Steven Liu

**Affiliations:** ^1^ University of South Carolina Columbia South Carolina USA

**Keywords:** bias, bootstrap, Fisher's exact test, odds ratio

## Abstract

**Objectives:**

The odds ratio estimate in Fisher's exact test can overestimate the parameter. A simple computer simulation can easily reveal the positive bias of the odds ratio estimate from Fisher's exact test. Bootstrap can facilitate bias correction for the odds ratio estimate.

**Methods:**

The bias can be estimated, using bootstrap samples and the original sample to approximate the expectation of the odds ratio estimator and the true parameter value—their difference is the bias. Here, the bias is computed from the underlying distribution, conditional on the exclusion of zero cells in sampling, to avoid the infinite expectation.

**Results:**

A study of depression is used to demonstrate how to use bootstrap to correct the bias in an odds ratio estimate based on Fisher's exact test.

**Conclusions:**

Bootstrapping can easily estimate and correct the bias of an odds ratio estimate in Fisher's exact test. The results suggest that bootstrapping is sensitive enough to detect even a small bias.

## 1 Introduction

Odds ratio is a popular index to show the strength of association between two binary variables in medical research. One binary variable can be disease status, and the other can be a risk or exposure factor. For example, the disease can be depression, and the risk factor can be disruptive life event (e.g., death in a family, job loss, divorce, etc.). An odds ratio is based on the idea of odds, which is the probability of one event divided by the probability of the opposite event, that is, the probability of having depression over the probability of no depression. Unlike a probability that is confined to 0 and 1, an odds can range from zero to positive infinity. The elongated range makes it easy to compare the probabilities of a certain event between two groups (i.e., the groups with and without the risk factor). Suppose that the risk group has a 0.60 probability of having depression, and that the group without the risk has a 0.30 probability of having depression. The odds of depression for the group with the risk is 0.60/(1 − 0.60) = 1.5, and the odds of depression for the group without the risk is 0.30/(1 − 0.30) = 0.43. The odds ratio of depression between the two groups is 1.5/.43 = 3.5, so the risk increases the odds of depression by 3.5 times.

Data arising from odds ratio are often presented in a two‐way contingency table with counts in the four table cells. The counts in the two‐way contingency table can be used to estimate the odds ratio. There are a few ways to estimate the odds ratio using data in the two‐way contingency table. The simplest way is to use counts in the two‐way table (see Table 1). The counts a, b, c, and d represent the number of cases in each combination of the two binary variables. The odds ratio (OR) is calculated as OR=(ad)/(cb). Specifically, sample proportions are used to estimate the corresponding probabilities in the odds ratio. In the risk group, the probability of disease is based on the proportion of disease, that is, a/(a + b), so the probability of no disease in the risk group is b/(a + b). The odds of disease for the risk group is therefore a/b. Likewise, the odds of disease in the group without risk is c/d. The ratio of the two odds gives a simple estimate of odds ratio, (ad)/(cb). It should be noted that the empirical odds ratio can take the value of infinity for zero cells, so an infinite expectation may occur in sampling, albeit with small probability. Nonetheless, this difficulty with infinity odds ratio can be addressed by taking the expectation, conditional on the exclusion of zero cells (Jewell 1986).

**TABLE 1:** Two‐way contingency table.

	Disease	No disease
Risk	a	b
No risk	c	d

The estimates of odds ratio can also be obtained from two common statistical procedures: one is the logistic regression and the other Fisher's exact test. Note that these two statistical procedures use different ways to estimate the odds ratio. Logistic regression features a binary outcome and continuous and/or categorical predictors including a binary predictor, so it is applicable to data analysis of a two‐way contingency table. In the case of a binary outcome and a binary predictor, the regression coefficient of the binary predictor can be used to estimate the odds ratio, that is, the exponential function of the logistic regression coefficient is the odds ratio. It literally means a multiplicative change in the odds of the binary outcome being one if the binary predictor goes from zero to one. Also, the logistic regression analysis allows testing the regression coefficient and, in turn, the odds ratio. The significance test uses a chi‐square or Wald statistic, based on asymptotical large sample approximation. The Wald test makes it feasible to identify statistical significance in declaring association between two binary variables, which adds to its popularity in data analysis of a two‐way contingency table for publication. However, the *p*‐value for the significance test in logistic regression is approximate but not exact. That is where Fisher's exact test becomes a nice alternative.

Fisher's exact test is preferred in analyzing a two‐way contingency table with limited sample size. The test is credited to Fisher, who originally devised an experiment to examine a lady's claim whether she knew tea or milk poured first in a cup of tea with milk (Fisher 1935). Eight cups of tea with milk were presented to the lady. Four cups had milk poured first, and the other four had tea poured first. The lady tasted eight cups of tea and rendered her opinions. The results were compiled in a two‐way contingency table. Fisher used hypergeometric distribution to derive the *p*‐value of the statistic, which basically tested the odds ratio being one in the null hypothesis. The exact *p*‐value is an advantage in significance test because large sample approximation like chi‐square performs poorly in a small sample. It should be noted that exact *p*‐value is just one consideration when deciding a test, and that there are generally other considerations, including Type I error, statistical power, and their trade‐off. As Fisher's exact test is often the choice of analysis for a two‐way table, the current paper will focus on the odds ratio estimate used in Fisher's exact test.

The odds ratio estimate in Fisher's exact test is a “conditional ML estimate” that maximizes the likelihood function of the observed count. Specifically, Fisher's conditional maximum likelihood estimate uses a likelihood function, based on a non‐central hypergeometric distribution. For brevity of presentation, let θ be the parameter of the odds ratio in the non‐central hypergeometric distribution. The probability mass function of the non‐central hypergeometric distribution is as follows (Agresti 2002, eq. 3.20, p. 99):

$$P_{\theta }\left(A=a|R_{1},C_{1}\right)=\frac{\left(\begin{array}{c} C_{1}\\ a \end{array}\right)\left(\begin{array}{c} C_{2}\\ R_{1}-a \end{array}\right)\theta^{a}}{\sum_{k_{\min}}^{k_{\max}}\left(\begin{array}{c} C_{1}\\ a \end{array}\right)\left(\begin{array}{c} C_{2}\\ R_{1}-k \end{array}\right)\theta^{k}}$$

where a is the observed A, $k_{\min}$ and $k_{\max}$ are the bounds of a, $C_{1}$ is the column margin ($C_{1}=a+c$), and $R_{1}$ is the row margin ($R_{1}=a+b$). Differentiating the log likelihood with respect to θ shows that the estimate satisfies the equation, $E_{\theta }\left(A\right)=a$. This equation has a unique solution $\hat{\theta }$ (i.e., conditional ML estimate) and is solved iteratively. The conditional ML estimate $\hat{\theta }$ is different from the unconditional ML estimator, (ad)/(cb) (Agresti 2002, para. 2, p.100). The unconditional ML estimate is often called simple odds ratio estimate. Although the conditional ML estimate and simple odds ratio estimate are often very close, they can be quite different in some circumstances. For instance, when *a* = 15, *b* = 4, *c* = 5, and *d* = 26, the simple odds ratio estimate, (ad)/(bc), is 19.5, and the odds ratio estimate from Fisher's exact test is 17.9, about 8% smaller.

The Fisher's exact test and its *p*‐value can be exactly derived, so is the confidence interval for the odds ratio estimate. This has added greatly to the popularity of Fisher's exact test in analyzing the data from a two‐way contingency table. The statistical literature also suggests that Fisher's exact test is favorable, compared to other alternative procedures. Hauck (1984) found that the conditional maximum likelihood estimate was superior to other estimates, including unconditional maximum likelihood estimate, in terms of bias and precision. Bind and Rubin (2020) showed that asymptotic *p*‐value based on large sample approximation should not always be trusted. The asymptotic *p*‐value depended on unattainable null distribution, which could effectively change the research question. They recommended using the exact *p*‐value in Fisher's exact test, instead of the commonly reported asymptotic *p*‐value.

Although the *p*‐value from Fisher's exact test is exact, the odds ratio estimate used in Fisher's exact test is not unbiased. The bias of an estimator ($\hat{\theta }$) is defined as the difference between its expectation and the parameter, that is, $bias=E\left(\hat{\theta }\right)-\theta$. Subtracting the bias from a biased estimator yields a bias corrected estimator ${\hat{\theta }}_{c}=\hat{\theta }-bias$. The bias corrected estimator ${\hat{\theta }}_{c}$ is unbiased because its expectation is equal to the parameter, θ. In this paper, we will first show the bias and then try to estimate the bias in correcting the biased estimator. It will be tedious, if not impossible, to analytically derive the bias for the odds ratio in Fisher's exact test. The mathematics involved can quickly become intractable. Given today's computing technology, a simple computer simulation can be readily utilized to demonstrate the bias in the odds ratio estimate from Fisher's exact test. The implementation of the simulation is easy for practitioners to understand, and the results are straightforward to interpret.

## 2 Bias of Odds Ratio Estimate

Computer simulations can be devised to reveal the bias in odds ratio estimate from Fisher's exact test. The computer simulation below covers three different odds ratios that correspond to Cohen's small, medium, and large effect sizes in a two‐way contingency table. Cohen created a general effect size *w* to measure the association between two categorial variables in a two‐way contingency table. The effect size *w* represents the discrepancies in proportion between the null hypothesis and the alternative hypothesis. To provide some guidance on the magnitude of the effect size *w*, Cohen used 0.10, 0.30, and 0.50 for small, medium and large effect size (Cohen 1988, 224–225). It should be noted that these rule‐of‐thumb numbers on effect size *w* need to be treated with caution in the context. As effect size measures can dramatically vary from one field to another, the rule‐of‐thumb numbers should be adjusted accordingly with reference to its practical importance in the context. In the case of a 2 × 2 contingence table there are only four proportions. Cohen (1988, 223) provided a formula to convert the chi‐square statistic in the contingency table to the effect size *w*, namely, $w=\sqrt{\chi^{2}/N}$ , where $\chi^{2}$ is the chi‐square statistic for the contingency table, and N is the total sample size. Using this formula, we produce three hypothetical populations of size 10,000 each (N=10,000) to represent Cohen's effect size (*w*) 0.10, 0.30, and 0.50. The population odds ratio can be calculated from the population proportions in the two‐way table, using the formula OR=(ad)/(cb). The counts in the 2 × 2 table can be used to calculate the true proportions in the small population.

In the computer simulation a sample of 100 is repeatedly drawn without replacement from a finite population, say, 10,000 subjects. The population size 10,000 is sufficiently large and is chosen such that it renders the proportions in a 2 × 2 table obvious. Each sample returns an odds ratio estimate from Fisher's exact test. The mean of the odds ratio estimates is then compared to the known odds ratio for the finite population. Any discrepancy between the mean of the odds ratio estimates and the known population odds ratio is the bias in odds ratio estimate from Fisher's exact test. Tables 2, 3, 4 show the counts in the 2 × 2 contingency tables, and they closely match Cohen's *w* 0.1, 0.3, and 0.5. The R code in the appendix is used to generate repeated samples of 100 from a finite population of 10,000. The odds ratio estimates from Fisher's exact test are retained to compute the expectation or mean of the estimates.

**TABLE 2:** Two‐way contingency table for Cohen's w 0.1.

	Disease	No disease
Risk	2650	2150
No risk	2350	2850

**TABLE 3:** Two‐way contingency table for Cohen's w 0.3.

	Disease	No disease
Risk	2870	1430
No risk	2130	3570

**TABLE 4:** Two‐way contingency table for Cohen's w 0.5.

	Disease	No disease
Risk	3200	800
No risk	1800	4200

Table 2 produces an odds ratio 1.49 in a finite population of 10,000. The corresponding Cohen's *w* is about 0.10 or a small effect size of association between two binary variables. Assume that a sample of 100 subjects are repeatedly drawn from the population. Each random sample returns an odds ratio estimate from Fisher's exact test procedure (see the R code in the appendix). The Fisher's exact test procedure uses the non‐central hypergeometric distribution to obtain the conditional ML estimate of odds ratio (Agresti 2002, 99, Eq. 3.20). This process is repeated 50,000 times. The number of resampling processes is subjective and is usually set to a large number (e.g., 50,000) to emulate a sampling distribution. The 50,000 odds ratio estimates will produce a sampling distribution, which has a mean 1.63. Since the parameter odds ratio of 1.49 is known in this finite population, the bias is the difference between the mean 1.63 and the parameter, that is, bias = 1.63–1.49 = 0.14. Similarly, Table 3 yields a population odds ratio 3.36 and Cohen's *w* 0.29. The mean of the sampling distribution of odds ratio estimates from Fisher's exact test is 3.76, and the bias is 3.76–3.36 or 0.40. Table 4 represents a population odds ratio 9.33 and Cohen's *w* 0.49. The mean of the sampling distribution of odds ratio estimates from Fisher's exact test is 11.15, which indicates a positive bias of 1.82, that is, 11.15–9.33 = 1.82. The three simulations for Cohen's effect size *w* 0.1, 0.3, and 0.5 all point to positive bias in the odds ratio estimate from Fisher's exact test. The computer simulations basically confirm the existence of positive bias in odds ratio estimates from Fisher's exact test. Note that odds ratio is symmetric. For instance, the odds ratio for smokers compared to non‐smokers is 2. By symmetry, the odds ratio for non‐smokers compared to smokers will be 1/2 (or 0.5). Its estimation bias would be in the opposite direction. It is worth noting that the magnitude, and not just the direction, of the bias in the second case is different from that in the first case.

The bias of the odds ratio estimate can be undefined or infinite when either cell *c* or *b* in Table 1 is zero (Jewell 1986). In this situation, common practice is adding 0.5 to each cell in the two‐way table to resurrect the odds ratio estimation (Agresti 2019, 230). Without adding 0.5 to cells, the estimation will not work, due to a zero denominator. Adding 0.5 to cells can also improve confidence interval coverage (Agresti and Min 2005), although this is not generally used as a bias correction procedure. Additionally, adding 0.5 does not work with Fisher's exact test because it requires whole numbers (integers) as input. In Fisher's conditional maximum likelihood estimation, the probability mass function of the non‐central hypergeometric distribution uses integers for the margins. Becker (1989) treats all two‐way tables with possible zero cells as “unconditional sample space” and tables with non‐zero cells as “conditional sample space”. The latter case, for practical purposes, covers most situations and is the focus of the current paper. Jewell (1986) suggests adding 1 to cell *c* and *b* to mitigate the bias of odds ratio estimate in small samples, but Becker (1989) disagrees with Jewell's bias correction. Readers can refer to the letter to the editor in *Statistics in Medicine* (Jewell 1991) for exchange of ideas on this issue between the two scholars. As Jewell's bias correction is intended for the unconditional ML estimate of odds ratio, no further discussion will be extended here, so the focus of the current paper will be on bias correction of conditional ML estimate of odds ratio.

In view of the literature (Jewell 1986), the current study will only use tables with non‐zero cells. In fact, Jewell (1986, 353) excludes zeros in bias estimation for odds ratio and remarks about discarding zeros in the following excerpt:

The exact bias of $\hat{OR}$ is infinite due to the fact that the cell values b or c can be zero with nonzero probability. To compare the small‐sample bias of $\hat{OR}$ and ${\hat{OR}}_{ss}$ it is thus appropriate to perform the calculations conditional on the event that neither b nor c is zero. The exact bias of $\hat{OR}$ and ${\hat{OR}}_{ss}$ was computed for a variety of p1, p2 in cohort sampling, conditional on neither b nor c being zero.

Thus, zero counts are discarded for practical purposes. It is a reasonable thing that someone will do with the sample. Just imagine that b is 0 in a two‐way table, which means 0 subject, disease free (cancer free) with exposure (smoking)—this sample, if standalone, will be discarded, as infinity means causation. It defies the purpose of a two‐way table, which under normal circumstances is about association, not causation. It should be noted that zero counts are possible in a large number (10,000) of simulated samples, but are very rare by frequency of occurrence. In a smaller number of simulated samples, say a few hundred, they may not occur at all. In other words, zero counts can be avoided with limited simulations, so readers should be aware that the methodology is limited to non‐zero cells in the two‐way table, and that the result is conditional on excluding zero cell counts. For most practical purposes, it is, nonetheless, appropriate to keep the large number of simulated samples and discard a few zero counts because infinity bias is not automatically considered in most circumstances.

## 3 Bootstrap Bias Correction

Bootstrap bias correction offers a simple solution to correcting the bias in the conditional ML estimate of odds ratio, that is used in Fisher's exact test. Bootstrap bias correction has two unique advantages over other alternative procedures. First, bootstrap does not make strong distribution assumptions about the data. All the analytical formulas for bias correction assume certain distributions, which are not attainable in small studies with limited data. Second, the bootstrap bias correction uses the same procedure, regardless of the estimation method. It does require a lot of computing power, but it no longer poses any challenge, given today's computer speed. The involved computation can often be done in a little time with open‐source software (e.g., R).

In bootstrap bias correction, the estimates are repeatedly calculated from bootstrapped samples of the same size, which are random samples from a population. Since the population is not entirely accessible, the original data substitutes as a population. The original estimate from the original data, therefore, becomes the surrogate parameter because it is now viewed as coming from a population. Given the population and parameter, bootstrapping allows us to see how the estimate behaves in the bootstrapped samples. Each bootstrapped sample is a random sample of the same size from the original data, and each bootstrapped sample yields an estimate. The estimates from all the bootstrapped samples reveal how estimates perform in relation to its parameter, which is the original estimate from the original data. Those estimates from bootstrapped samples are called bootstrap replicates, which together approximate the sampling distribution of the estimates. If the expectation of the sampling distribution of the estimates coincides with the original estimate from the original data, it suggests that estimates from bootstrapped samples on average equal its parameter: No bias exists. Any discrepancy between the mean of bootstrap replicates and the original estimate indicates the existence of bias, and their difference is the estimated bias, which can be used to correct the estimate (Efron 1979; Efron and Tibshirani 1993).

Suppose that $OR_{0}$ represents the original odds ratio estimate in Fisher's exact test for the original data, from which random samples of the same size are drawn in the bootstrapping procedure. Each bootstrapped sample produces an odds ratio estimate based on Fisher's exact test, which is denoted by $OR_{i}$ (i.e., the *i*th bootstrap replicate). There are B number of bootstrapped samples and *B* number of bootstrap replicates. The mean of all the bootstrap replicates is compared with the original estimate $OR_{0}$. Their difference is the estimated bias ($\hat{bias}$), that is, $\hat{bias}=\frac{\sum_{i=1}^{B}OR_{i}}{B}-OR_{0},$ where B is the number of bootstrap repetitions, and $OR_{i}$ is the estimate from Fisher's exact test for the *i*th bootstrapped sample. Therefore, the bias‐corrected estimate $OR_{c}$ is two times the original estimate minus the mean of all bootstrap replicates.

$$OR_{c}=OR_{0}-\hat{bias}=2\times OR_{0}-\frac{\sum_{i=1}^{B}OR_{i}}{B}$$

## 4 Example

The data on depression and peer victimization came from the Avon longitudinal study of parents and children that included 14,000 children born between 1991 and 1992 in England. The longitudinal study examined a variety of problems in children's health and development. The data used here investigated the relationship between being bullied at age 13 and depression at age 18. The total sample size was 3898 (Moore et al. 2021). The subjects were classified by being bullied and depression in a 2 × 2 contingency table (see Table 5).

**TABLE 5:** Depression and bully.

	Depressed	Not depressed
Bullied	204	1925
Never bullied	97	1762

**FIGURE 1:**
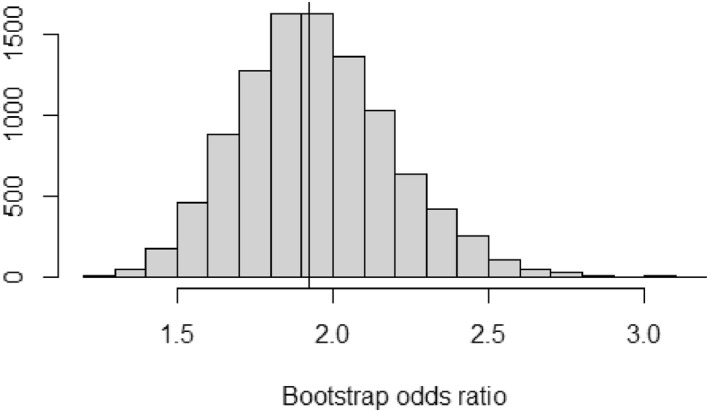
Sampling distribution of bootstrapped odds ratio estimates.

The odds ratio of depression between being bullied and not being bullied is in question. The odds ratio estimate in Fisher's exact test is $OR_{0}=1.9246$. Knowing the existence of positive bias, bootstrap is deployed to estimate the bias in the odds ratio estimate. The number of bootstrap repetitions is set to 50,000 (see the R code in the appendix). Note that bootstrap samples with zero cells are dropped to control for undefined or infinite bias. The histogram of bootstrap replicates in Figure 1 shows a slightly right‐skewed distribution of bootstrapped odds ratio estimates. The vertical line in the histogram represents the odds ratio estimate from Fisher's exact test, 1.925. The mean of bootstrap replicates is $\sum_{i=1}^{B}OR_{i}/B=1.946$. So, bootstrapping indicates a bias of 0.02, that is, $\hat{bias}=1.946-1.925=0.02$. The bias‐corrected odds ratio estimate is, therefore, $OR_{c}=1.90$. It should be noted that the odds ratio of 1.90 is close to a small‐sized odds ratio 1.46 that corresponds to Cohen's small effect size w=0.10. Bootstrap appears to be sensitive enough to detect even a small bias in the odds ratio estimate from Fisher's exact test.

## 5 Conclusion

The odds ratio estimate from Fisher's exact test has a positive bias, which can be easily verified in computer simulations. There are many factors that can contribute to the bias in odds ratio estimate, so it is almost impossible to analytically track and correct the bias in the odds ratio estimate. The involved mathematics can quickly become intractable. Besides, analytical solutions to bias require distributional assumptions that are often not tenable in small studies with limited data. Bootstrap offers a simple computational solution to estimating and removing the potential bias in an odds ratio estimate from Fisher's exact test. The limitation of bootstrap bias correction, though, is zero cell counts in the two‐way table, which can result in infinity bias estimate. Excluding zero cell counts, which is used in Jewell (1986), is not a perfect solution, but it functions well for most practical purposes. Although Fisher's exact test is widely used to analyze data in a two‐way contingency table, the bias of its odds ratio estimate is rarely corrected in publication. Using simple R code, we have demonstrated that bootstrap can easily estimate and correct the bias. The used example suggests that bootstrapping is sensitive enough to detect even a small bias in an odds ratio estimate.

## Author Contributions

**Xiaofeng Steven Liu:** conceptualization, methodology, software, data curation, investigation, validation, formal analysis, supervision, funding acquisition, visualization, project administration, resources, writing – original draft, writing – review and editing.

## Ethics Statement

The author has nothing to report.

## Conflicts of Interest

The author declares no conflicts of interest.

## Data Availability

Data sharing not applicable to this article as no datasets were generated during the current study.

## R Code

#simulate bias in OR from Fisher's exact test
#Cohen w 0.10 OR 1.49
*a* = 2650
*c* = 2350
*b* = 2150
*d* = 2850
#Cohen w 0.29 OR 3.36
*a* = 2870
*c* = 2130
*b* = 1430
*d* = 3570
#Cohen w 0.49 OR 9.33
*a* = 3200
*c* = 1800
*b* = 800
*d* = 4200
tb = matrix(c(a,c,b,d),nrow = 2)
or = (a * d)/(c * b)
or
chisq = chisq.test(as.table(tb))$statistic
x2 = unname(chisq)
sqrt(x2/10000) #Cohen's w or phi
#use the following code to calculate bias
or_est = numeric()
for(i in 1: 50000){
 disease_i = rhyper(1,a,c,50)
no_disease_i = rhyper(1,b,d,50)
 a_i = disease_i
 b_i = no_disease_i
 c_*i* = 50‐a_i
 d_*i* = 50‐b_i
 if(a_i ! = 0 & b_i ! = 0 & c_i ! = 0 & d_i ! = 0){
or_i = unname(fisher.test(matrix(c(a_i,c_i,b_i,d_i),
nrow = 2))[[3]])
 or_est = c(or_est, or_i)
 }
}
bias = mean(or_est)‐ or #bias
#bootstrap bias correction: data from Moore BPS 9 ed. p. 559
*a* = 204
*b* = 1925
*c* = 97
*d* = 1762
or_ml = unname(fisher.test(matrix(c(a,c,b,d),nrow = 2))[[3]])
depress = c(rep(1,a),rep(0,c))
no.depress = c(rep(1,b),rep(0,d))
or_ml_b = numeric()
for(i in 1:50000){
 depress_i = sample(depress, replace = TRUE)
 no.depress_i = sample(no.depress, replace = TRUE)
 a_i = sum(depress_i)
 b_i = sum(no.depress_i)
 c_i = (a + c)‐a_i
 d_i = (b + d)‐b_i
 if(a_i ! = 0 & b_i ! = 0 & c_i ! = 0 & d_i ! = 0){
or_ml_i = unname(fisher.test(matrix(c(a_i,c_i,b_i,d_i),nrow = 2))[[3]])
 or_ml_b = c(or_ml_b, or_ml_i)
 }
}
bias_ml = mean(or_ml_b)‐or_ml
or_ml‐bias_ml
